# A probable case of tick-borne encephalitis (TBE) acquired in England, July 2019

**DOI:** 10.2807/1560-7917.ES.2019.24.47.1900679

**Published:** 2019-11-21

**Authors:** Teresa M Kreusch, Maya Holding, Roger Hewson, Thomas Harder, Jolyon M Medlock, Kayleigh M Hansford, Stuart Dowall, Amanda Semper, Tim Brooks, Amanda Walsh, Katherine Russell, Ole Wichmann

**Affiliations:** 1 Immunization Unit, Robert Koch Institute, Berlin, Germany; 2 Virology and Pathogenesis Group, National Infection Service, Public Health England, Porton Down, United Kingdom; 3 NIHR Health Protection Research Unit in Emerging and Zoonotic Infections, Liverpool, United Kingdom; 4 Medical Entomology Group, Emergency Response Department, Public Health England, Porton Down, United Kingdom; 5 Tuberculosis, Acute Respiratory, Gastrointestinal, Emerging/Zoonotic Infections and Travel Health Division, National Infection Service, Public Health England, London, United Kingdom

**Keywords:** tick-borne encephalitis, case report, surveillance, England, Germany

## Abstract

The United Kingdom (UK) has thus far been considered to be free from tick-borne encephalitis (TBE), yet in July 2019, a German infant developed serologically diagnosed TBE following a tick bite in southern England. This first report of a probable human case together with recent findings of TBE virus in ticks in foci in England suggest that TBE may be acquired in parts of England and should be considered in patients with aetiologically-unexplained neurological manifestations.

End-July 2019, a case of tick-borne encephalitis (TBE) in a 3-month-old infant was notified to the German mandatory surveillance system for infectious diseases. The patient’s family, resident in a TBE-non-endemic region in Germany, had holidayed in England during the incubation time. We present the case report based on German surveillance data, information provided by the family, laboratory reports and two hospital discharge summaries, and describe the public health response.

## Case report

**Figure 1:**
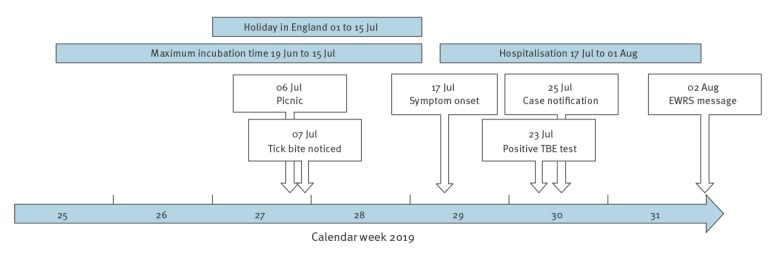
Timeline of infection, disease progression and public health response to the probable tick-borne encephalitis case in an infant, Germany, July–August 2019

EWRS: Early Warning and Response System; TBE: tick-borne encephalitis.

**Figure 2:**
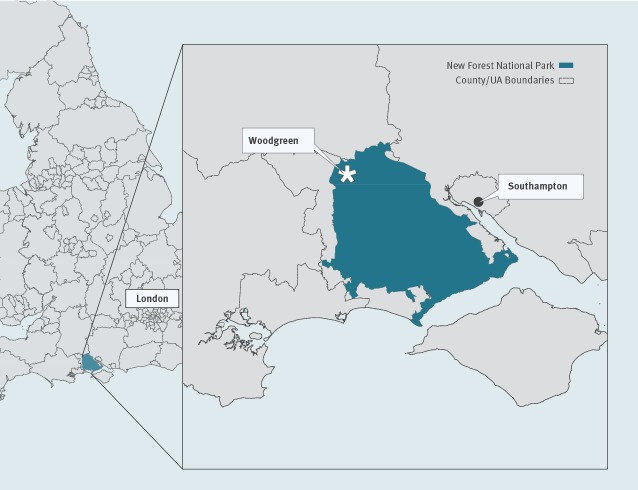
Map of the likely place of infection of tick-borne encephalitis case in a German infant, Woodgreen, New Forest National Park, England, 2019

TBEV: tick-borne encephalitis virus; UA: unitary authority.

Source: Ordnance Survey and National Statistics data for geographical and administrative boundaries.

A German family including a 3-month-old infant spent their holiday in southern England from 1 to 15 July 2019 (Figure 1). The mother was not vaccinated against nor reported past TBE infection. On 6 July, the family picnicked near Woodgreen in the New Forest National Park (Figure 2), where the child laid on a blanket on the grass. An unengorged tick, attached to the infant’s neck, was discovered on 7 July. The tick was removed incompletely, using tweezers, and the wound was disinfected. The remaining tick fragments detached 2 days later.

The previously healthy infant developed fever on 17 July, 11 days after the tick bite. Medical history was unremarkable; the infant had thus far received one hexavalent routine childhood vaccination. Subtracting the maximum incubation period of 28 days [1] from symptom onset, renders 19 June as the earliest possible infection date. The infant reportedly did not visit any other location where a tick bite could have occurred except their home area in Hesse, Germany which is non-endemic for TBE. Each bout of fever was accompanied by focal seizures, lasting ca 1 min. Hospitalisation occurred on 17 July, prompting a series of diagnostic tests (Table). Based on elevated cerebrospinal fluid markers (Table), meningitis was diagnosed and the infant was treated with intravenous cefotaxime, ampicillin and aciclovir. The focal seizures became generalised lasting up to 5 min and were treated with anticonvulsants (clonazepam, midazolam, levetiracetam). The infant was transferred to a specialised hospital on 20 July. Magnetic resonance imaging and repeated electroencephalograms revealed pathological results (Table). Having excluded numerous neurotropic pathogens, TBEV-specific serology tested positive for IgM and IgG (Table) and meningoencephalitis because of TBEV infection was diagnosed by the treating physicians. The infant was discharged 15 days after admission with mild remaining neurological symptoms, which had subsided by the check-up 6 weeks later.

**Table:** Diagnostic tests performed on the probable case of tick-borne encephalitis (TBE) during hospitalisation, Germany, July–August 2019

Date (2019)	Test (sample type/assay)	Result	Interpretation
17–19 Jul^a^	CSF diagnostics	1,000 cells (norm: 0–5) (40% granulocytes, 60% lymphocytes) 1.5 g protein 59 mg/dL glucose level (norm: 40–80)	Inflammation
17–19 Jul^a^	Blood culture	Negative	Normal
17–19 Jul^a^	CSF culture	Negative	Normal
17–19 Jul^a^	Multiplex PCR (CSF) for: -*Escherichia coli*, -*Haemophilus influenzae*, -*Listeria monocytogenes*, -*Neisseria meningitidis*, -Streptococcus, -Cytomegalovirus, -Enterovirus, -Herpes Simplex Virus 1 and 2, -Human herpesvirus 6, -Human parechovirus, -Varicella zoster virus, -Cryptococcus	Negative	Normal
17–19 Jul^a^	Enterovirus (stool)	Negative	Normal
20 Jul	MRSA and MRGN screening	Negative	Normal
23 Jul	TBEV-IgG (serum)^b^	10.3 AE/mL	Positive (cut-off: 0.241)
23 Jul	TBEV-IgM (serum)^b^	12.5 index	Positive (cut-off: 0.234)
25 Jul	*Borrelia burgdorferi* (IgG-ELISA)	< 5.2 U/mL	Negative (cut-off: < 7 U/mL)
22 Jul and 25 Jul	Electroencephalography	Slowed activity in right hemisphere; Epileptic activity in right temporal/central and left occipital areas.	Pathological
01 Aug	Magnetic resonance imaging	Leptomeningeal enhancement. No sign of parenchymal defect or brain abscess.	Pathological

CSF: cerebrospinal fluid; MDRGN: multidrug resistant Gram-negative bacteria; MRSA: methicillin-resistant *Staphylococcus aureus*; TBEV: tick-borne encephalitis virus.

^a^ Performed during the first hospital stay. Exact test dates were not given in discharge summary.

^b^ The test kit used was Enzygnost Anti-TBE virus (IgG, IgM) (Siemens, Marburg, Germany) which determines a specific cut-off for each run (alpha-method).

## Public health response

Upon receiving the notification on 25 July, the Robert Koch Institute asked the patient’s family for their detailed travel history in England. One week later, the event was reported through the European Commission Early Warning and Response System (EWRS) selective exchange to inform United Kingdom (UK) colleagues.

Following TBEV detection in ticks in Thetford Forest in 2019, from samples collected February 2018 to January 2019 [2], enhanced clinical surveillance activities were underway in the east of England, focusing on encephalitis cases without confirmed cause [3]. Following the EWRS message, these activities were extended to areas surrounding the New Forest National Park. TBEV seroprevalence studies in groups at high risk of tick bites and in the general population are also being implemented in both areas. Tick surveillance was already underway around the New Forest National Park following previous findings [2], but additional tick surveys were conducted around Woodgreen on 8 and 23 August 2019. Only 135 ticks (70 nymphs, 25 adult males, 40 adult females) were collected, likely because the peak tick questing season had already passed. Pools of 10 nymphs, five adult males or five adult females were homogenised for RNA extraction and RT-PCR analysis [4]. No TBEV or other TBEV-serocomplex RNA was detected.

## Discussion

We report a human TBE case, believed to be the first acquired in the UK. Diagnosis was by serology only, which can be regarded as a limitation. No reserve sample was available for additional testing (TBE-specific PCR or neutralization assay [5]). Because of the lack of therapeutic consequences, no follow-up blood sample was drawn from the infant; therefore it was not possible to test for a rise in IgG titre in paired samples [5].

Several pieces of evidence support the likelihood that this is a true TBE case. First, the tick bite, the clinical symptoms and the incubation time of 11 days, close to the median of 8 days [5], fit the typical picture of TBEV-infection. This patient did not have the biphasic course of TBE, which is observed in 72–87% of TBE cases [5]. Second, as the infant resides in a TBE-non-endemic area in Germany it is highly unlikely that a second tick bite occurred there within the incubation time, went unnoticed and caused the infection. The likelihood of the infection having occurred near Woodgreen is far higher given the known tick bite. Third, the extensive array of differential diagnostics ruled out numerous other neurotrophic pathogens. Fourth, the TBE serological results were far above the cut-offs. Finally, as the mother did not report any past TBE vaccination or infection, it is unlikely that maternal TBE antibody transfer occurred, and certainly not with such high titres.

In 2019, TBEV was reported for the first time in ticks in discrete foci in Thetford Forest, England [2], but the pathogenicity is unknown and no other human cases have yet been identified in the UK. Tick surveys around Woodgreen did not detect any TBEV, however, it must be noted that only a small tick sample was collected. Yet, a pool of questing ticks sampled previously, on the Hampshire/Dorset border in June 2019, tested TBEV-positive, suggesting that TBEV has established itself in the UK [6]. Follow-up tick surveys will be conducted during spring 2020.

Although the clinical presentation and serology are consistent with the European TBE case definition [7], this interpretation has to be considered in light of the natural endemicity of Louping ill virus (LIV) in the UK. Until recently, LIV was believed to be the only virus of the TBE-serocomplex endemic in the UK [8]. Like TBEV, LIV is also transmitted by *Ixodes ricinus* ticks and mainly occurs in sheep, cattle and red grouse in upland grazing areas of the British Isles [8]. LIV infects humans in rare cases and cross-reacts with TBEV serologically. In the absence of an isolate or sequence data from acute phase samples, the exact aetiology in the case presented here remains uncertain. However, LIV is most prevalent in upland areas, which are located mostly in the north and west of the UK, and less than 50 human clinical LIV cases have been reported since 1934 [8], with one in England reported as recently as 2011 [9]. The likelihood of LIV thus is low in our case and we believe that it is a true TBEV-infection.

This first probable human TBEV-infection in England and the detection of TBEV in ticks stand in accordance with the patchy spread of TBEV to new areas observed in parts of Europe. In Germany, the number of TBE-endemic districts increased from 129 in 2007 to 161 in 2019 [10]. The first TBE cases from the Netherlands were reported in 2016 [11,12]; and a new focus was recently discovered in Denmark following three human TBE cases in summer 2019 [13]. TBEV can spread to new areas through mammalian hosts or migratory birds infested with TBEV-carrying ticks [14]. This may either lead to sporadic infections, or sometimes to the establishment of new foci, if local climatic conditions are favourable to the transmission cycles between ticks and their rodent hosts [5].

In England, the public health authorities currently assess the risk of TBEV infection as very low for the general population and low for those who may be bitten by ticks in areas where infected ticks are located [15]. Seroprevalence studies in groups at high risk of tick bites and in the general population, tick sampling and enhanced surveillance of human encephalitis cases without confirmed cause are underway to better understand the human infection risk in areas where TBEV was detected in ticks or wildlife. Public Health England continues to promote tick awareness for those spending time outdoors. The public health risks from TBEV in England will be dynamically reviewed as new findings come to light.

## References

[1] Kaiser R The clinical and epidemiological profile of tick-borne encephalitis in southern Germany 1994-98: a prospective study of 656 patients. Brain. 1999;122(Pt 11):2067-78. 10.1093/brain/122.11.2067 10545392

[2] Holding M Dowall SD Medlock JM Carter DP Pullan ST Lewis J Tick-Borne Encephalitis Virus, United Kingdom. Emerg Infect Dis. 2020;26(1). 10.3201/eid2601.191085 31661056 PMC6924911

[3] Public Health England (PHE). PHE briefing note 2019/40: Tick-borne encephalitis virus in the UK. [internal document]. 8 Aug 2019.

[4] Schwaiger M Cassinotti P Development of a quantitative real-time RT-PCR assay with internal control for the laboratory detection of tick borne encephalitis virus (TBEV) RNA. J Clin Virol. 2003;27(2):136-45. 10.1016/S1386-6532(02)00168-3 12829035

[5] Lindquist L Vapalahti O Tick-borne encephalitis. Lancet. 2008;371(9627):1861-71. 10.1016/S0140-6736(08)60800-4 18514730

[6] Holding M Dowall SD Medlock JM Carter DP McGinley L Curran-French M Detection of new endemic focus of tick-borne encephalitis virus (TBEV), Hampshire/Dorset border, England, September 2019. Euro Surveill. 2019;24(47):1900658. 10.2807/1560-7917.ES.2019.24.47.1900658 PMC6885748 31771701

[7] European Centre for Disease Prevention and Control (ECDC). EU case definitions: Tick-borne encephalitis. Solna; ECDC. [Accessed 5 Nov 2019]. Available from: https://www.ecdc.europa.eu/en/surveillance-and-disease-data/eu-case-definitions

[8] Jeffries CL Mansfield KL Phipps LP Wakeley PR Mearns R Schock A Louping ill virus: an endemic tick-borne disease of Great Britain. J Gen Virol. 2014;95(Pt 5):1005-14. 10.1099/vir.0.062356-0 24552787 PMC4811648

[9] Walkington J Hulgur M Yates D Eynon CA A case of refractory seizures caused by an unusual zoonosis. J Intensive Care Soc. 2013;14(1):65-6. 10.1177/175114371301400113

[10] Hellenbrand W Kreusch T Böhmer MM Wagner-Wiening C Dobler G Wichmann O Epidemiology of Tick-Borne Encephalitis (TBE) in Germany, 2001^−^2018. Pathogens. 2019;8(2):E42. 10.3390/pathogens8020042 30934855 PMC6630332

[11] de Graaf JA Reimerink JH Voorn GP Bij de Vaate EA de Vries A Rockx B First human case of tick-borne encephalitis virus infection acquired in the Netherlands, July 2016. Euro Surveill. 2016;21(33):30318. 10.2807/1560-7917.ES.2016.21.33.30318 27562931 PMC4998423

[12] Weststrate AC Knapen D Laverman GD Schot B Prick JJ Spit SA Increasing evidence of tick-borne encephalitis (TBE) virus transmission, the Netherlands, June 2016. Euro Surveill. 2017;22(11):30482. 10.2807/1560-7917.ES.2017.22.11.30482 28333618 PMC5356422

[13] Agergaard CN Rosenstierne MW Bødker R Rasmussen M Andersen PHS Fomsgaard A New tick-borne encephalitis virus hot spot in Northern Zealand, Denmark, October 2019. Euro Surveill. 2019;24(43):1900639. 10.2807/1560-7917.ES.2019.24.43.1900639 31662158 PMC6820129

[14] Hasle G Transport of ixodid ticks and tick-borne pathogens by migratory birds. Front Cell Infect Microbiol. 2013;3:48. 10.3389/fcimb.2013.00048 24058903 PMC3767891

[15] Human Animal Infections and Risk Surveillance group. HAIRS risk assessment: tick-borne encephalitis. Qualitative assessment of the risk that tick-borne encephalitis presents to the UK population. London: Public Health England; 2019. Available from: https://www.gov.uk/government/publications/hairs-risk-assessment-tick-borne-encephalitis

